# Biomechanical mechanisms of multidirectional dynamic compensatory muscle fatigue induced by abnormal cervical curvature: a cross-sectional case-control study based on surface electromyography and Cobb angle

**DOI:** 10.3389/fspor.2025.1704956

**Published:** 2025-12-04

**Authors:** Rui Yang, Mou Wang, Yifan Yang, Xingyu Zhang, Yiran Wu, Wenna Zhou, Tao Ding, Jing Xu

**Affiliations:** 1 School of Rehabilitation, Kunming Medical University, Kunming, China; 2 Department of Rehabilitation, The First Affiliated Hospital of Kunming Medical University, Kunming, China

**Keywords:** cervical lordosis, chronic neck pain, muscle function, surface electromyography, biomechanics

## Abstract

**Introduction:**

Cervical lordosis, a core indicator of spinal sagittal balance, is closely associated with cervical dysfunction when abnormal (straightening or kyphosis). The muscular system, including the upper trapezius (UT) and sternocleidomastoid (SCM), contributes to the mechanical stability of the cervical spine. However, how abnormal curvature affects dynamic muscle fatigue mechanisms remains unclear. This study investigated how abnormal curvature influences the performance of the UT and SCM during movement and their correlations.

**Methods:**

Employing a cross-sectional case-control design, 49 participants were enrolled. Cervical lateral radiographs were used to measure the C2-C7 Cobb angle, categorizing participants into an abnormal curvature group (25 chronic neck pain patients) and a normal curvature group (24 healthy individuals). Surface electromyography (sEMG) monitored the UT and SCM during six cervical movements at maximal voluntary contraction. Frequency-domain parameters analyzed included median frequency (MF), mean power frequency (MPF), and their slopes (MFs, MPFs).

**Results:**

The C2-C7 Cobb angle was significantly lower in the abnormal curvature group compared to the normal group (*p* < 0.01). During dynamic activities, muscle fatigue was exacerbated in the abnormal curvature group: during extension, the left UT showed significantly decreased MPF (*p* < 0.01) and MF (*p* < 0.05); during flexion, the left SCM exhibited significantly increased MPFs (*p* < 0.05) and MFs (*p* < 0.01). Correlation analysis revealed that within the abnormal curvature group, worsening kyphosis showed a weak positive correlation with the MPF of the right UT during right lateral flexion (*r* = 0.492, *p* < 0.05). Conversely, within the normal group, increasing lordosis correlated with reduced fatigue risk.

**Conclusions:**

Abnormal cervical lordosis significantly increases dynamic muscle fatigue, manifesting as deteriorated sEMG spectral characteristics in the UT and SCM. Furthermore, a complex relationship exists between the cervical Cobb angle and muscle fatigue. This confirms the hypothesis that abnormal curvature induces compensatory muscle activation. The findings provide a biological basis for rehabilitation strategies, suggesting that clinical interventions should be tailored based on cervical curvature type.

**Clinical Trail Registration:**

Chinese Clinical Trial Registry ChiCTR25001081490.

## 1 Introduction

Cervical Lordosis (CL) serves as a critical indicator for assessing sagittal spinal alignment (1), with its biomechanical properties closely associated with cervical dysfunction (2, 3). Over the past decade, research focus on cervical sagittal alignment has expanded from analyzing correlations with surgical outcomes (4, 5) to evaluating post-operative curvature changes following procedures such as laminoplasty (6) and cervical disc arthroplasty (7). With conceptual extensions from thoracolumbar sagittal studies, the cervical curvature assessment parameter system has progressively matured, incorporating novel metrics including the T1 sagittal (T1S) angle (8) and thoracic inlet angle (9), while further exploring their associations with health-related quality of life and cervical sagittal vertical axis (SVA) (10).

Although lordosis is considered the predominant natural physiological manifestation, up to 38.3% of asymptomatic individuals exhibit cervical kyphosis, with higher prevalence among younger populations (11). Cervical curvature abnormalities (e.g., kyphosis, straightened lordosis) not only increase biomechanical loading on the cervical spine (2) and reduce quality of life (11), but also impact global spinal balance by inducing compensatory changes in the thoracic-lumbar-pelvic complex (12).

Cervical stability primarily relies on two components: the muscular system and the osseligamentous system. Studies demonstrate that cervical muscles contribute up to 80% of mechanical stability, while the osseoligamentous system accounts for merely 20% (13). Concurrently, chronic neck pain (CNP) patients exhibit significantly greater muscle volume and fat infiltration in deep cervical extensors compared to healthy controls (14), with demonstrated causal relationships between cervical muscle strength imbalances and structural alterations (15). Clinical evidence confirms that in CNP patients, forward head posture shifts the head's center of gravity (increasing cervical flexion moment), compelling sustained contraction of the upper trapezius (UT) and sternocleidomastoid (SCM) to maintain postural equilibrium, thereby perpetuating a vicious cycle of muscle fatigue and pain (16, 17).

Despite the crucial role of muscular function in maintaining CL, current research exhibits significant limitations in mechanistic understanding, assessment methodologies, and technical applications. First, cervical compensatory mechanisms (e.g., adjacent segment compensation for kyphosis) remain unlinked to muscular dynamics. Second, assessment approaches predominantly focus on static postural muscle strength comparisons, lacking objective quantification of muscle fatigue during multi-directional movements (e.g., flexion, rotation). Third, while surface electromyography (sEMG) spectral parameters—such as median frequency (MF), mean power frequency (MPF), and their slopes (MFs, MPFs)—have demonstrated sensitivity in detecting muscle fatigue (18), they have not been systematically applied to evaluate populations with CL abnormalities.

Consequently, this study proposes the core hypothesis that abnormal CL induces compensatory activation pattern alterations in the UT and SCM during dynamic activities, manifesting as characteristic electromyographic spectral changes. To address this, a cross-sectional case-control design was implemented, pioneering the integration of C2–C7 Cobb angle measurements with multi-directional sEMG analysis. The objectives are threefold: to elucidate biomechanical compensation patterns in cervical musculature among individuals with abnormal curvature; to establish quantitative relationships between curvature parameters and electromyographic indicators; and to provide a theoretical basis for rehabilitation interventions targeting muscular training.

## 2 Materials and methods

This cross-sectional study enrolled patients with CNP and healthy controls. Participants were stratified into the Abnormal CL Group (CNP patients) and the Normal CL Group (healthy controls) based on cervical curvature status, with cervical muscle activity monitored using sEMG. Radiographic assessment of CL was performed using the C2–C7 Cobb angle. In the present study, the Abnormal Lordosis Group (cervical kyphosis or straightened curvature) was defined by a C2–C7 Cobb angle >−4° (19–22), while the Normal Lordosis Group (physiological lordosis) was defined by a C2–C7 Cobb angle ranging between −16° and −4°. Handedness was not considered in the participant selection criteria, and all participants were capable of performing activities of daily living.

### 2.1 Subjects

Sample size was calculated using G*Power software version 3.1.9.7 (23). Based on a pre-trial mean difference of 13.35 in muscle fatigue between the abnormal and normal lordosis groups, with an effect size *d* = 0.86, and assuming *α* = 0.05 (two-tailed test), power (1 – *β*) = 0.80, and equal sample sizes per group, the calculation indicated a minimum requirement of 22 participants per group (total *N* = 44). Ultimately, 49 participants were enrolled. Participants were recruited through the Rehabilitation Department of the hospital and media advertisements. All subjects exhibited no cervical instability; detailed inclusion/exclusion criteria are provided in Supplementary Material 1.

Prior to the experiment, all participants were fully informed of the study's purpose, procedures, and potential risks, and provided written informed consent. They were also explicitly advised of their right to withdraw at any time and were informed of the protocols for managing physical discomfort during the experimental procedures. This study protocol was strictly designed in accordance with the Declaration of Helsinki, approved by the Chinese Clinical Trial Registry (Number: ChiCTR2500108149; Date: 2025-08-26; accessible at http://www.chictr.org.cn/), and received ethical approval from the Institutional Review Board of the First Affiliated Hospital of Kunming Medical University (Approval Number: 2025L023). Considering the placement of electrodes on the sensitive cervical skin region, all participants underwent a preliminary electrode patch application experience prior to the formal testing.

### 2.2 Experimental protocol

Prior to testing, the investigator collected participants' general clinical data including age, height, and body weight, from which body mass index (BMI) was computed. All subjects were instructed to abstain from any cervical therapeutic interventions (including pharmacotherapy) for 24 h preceding the experiment.

Cervical lateral radiographs were obtained for all participants and stored in the clinical diagnostic imaging system (Neusoft PACS/RIS V5.5, Neusoft Group Co., Ltd.). The investigator performed two independent measurements of sagittal cervical parameters within this system, with the mean value recorded as the definitive C2–C7 Cobb angle.

**Figure 1:**
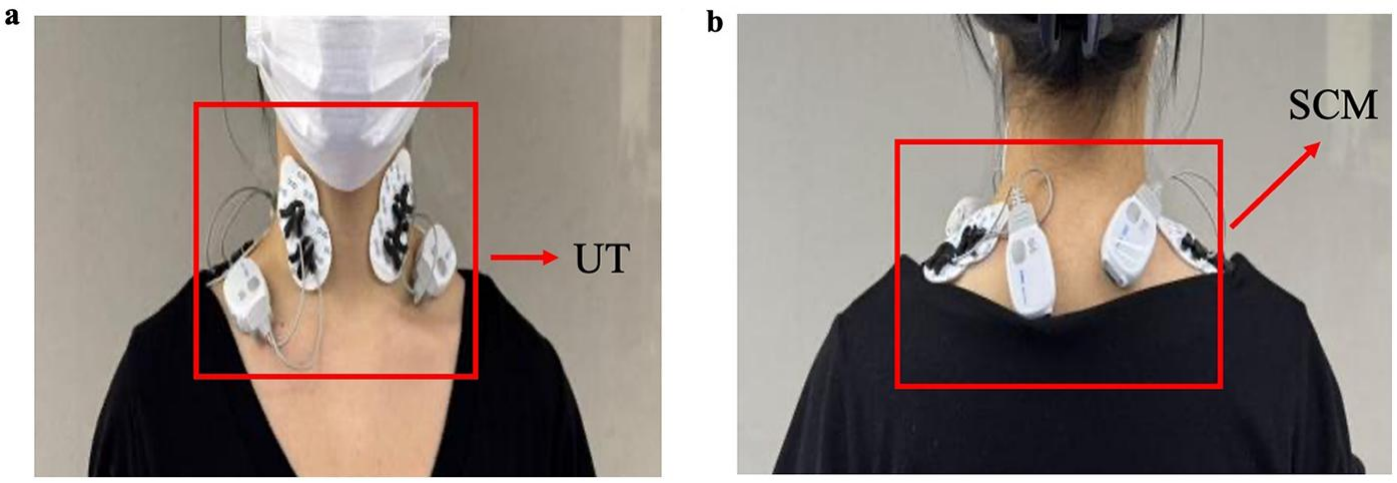
Depicts the electrode placement sites for surface electromyography. Panel **a** shows the electrode location at the UT, while **b** demonstrates the electrode placement on the SCM. UT, upper trapezius; SCM, sternocleidomastoid.

sEMG electrodes were positioned on the SCM and UT (Figure 1), selected for their critical role in cervical motion control (24) and suitability for sEMG due to their superficial topography and discrete anatomical boundaries (25). The testing protocol required participants to sequentially perform maximal isometric contractions in six cervical movement directions: flexion, extension, left lateral flexion, right lateral flexion, left rotation, and right rotation. Each direction was tested at maximal voluntary contraction intensity, sustained for 5 s, with three trials per direction. The mean EMG amplitude from the three trials was used for analysis. A 2-min rest interval was implemented between measurement sets to mitigate cumulative muscle fatigue effects.

### 2.3 Cobb angle measurement

**Figure 2:**
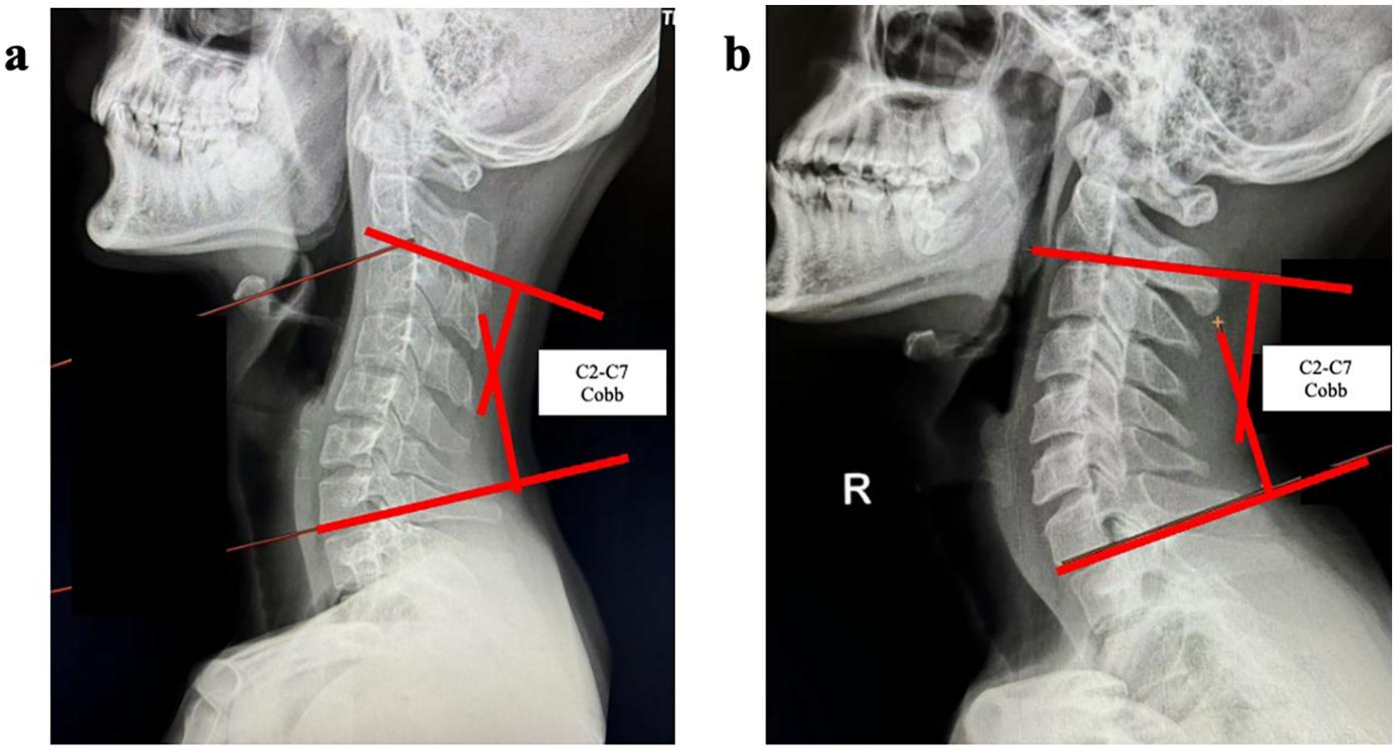
Cobb angle measurement. The **a** depicts the C2–C7 Cobb angle in a normal cervical lordosis and **b** demonstrates the C2–C7 Cobb angle in cervical kyphotic.

The C2–C7 Cobb angle defined as the angle between the lower plate (inferior endplate) of the second cervical vertebra and the lower plate (inferior endplate) of the seventh cervical vertebra (26). Cobb angle measurement was performed on cervical lateral radiographs. The C2 and C7 were identified radiographically, with C2 recognized by its distinctive odontoid process and C7 distinguished by its characteristically elongated and prominent spinous process on lateral views. Lines were drawn along the inferior endplate of C2 and the superior endplate of C7, maintaining parallelism to the respective endplates to the greatest extent possible. The angle subtended between these parallel lines defined the C2–C7 Cobb angle (Figure 2).

### 2.4 sEMG signal

**Figure 3:**
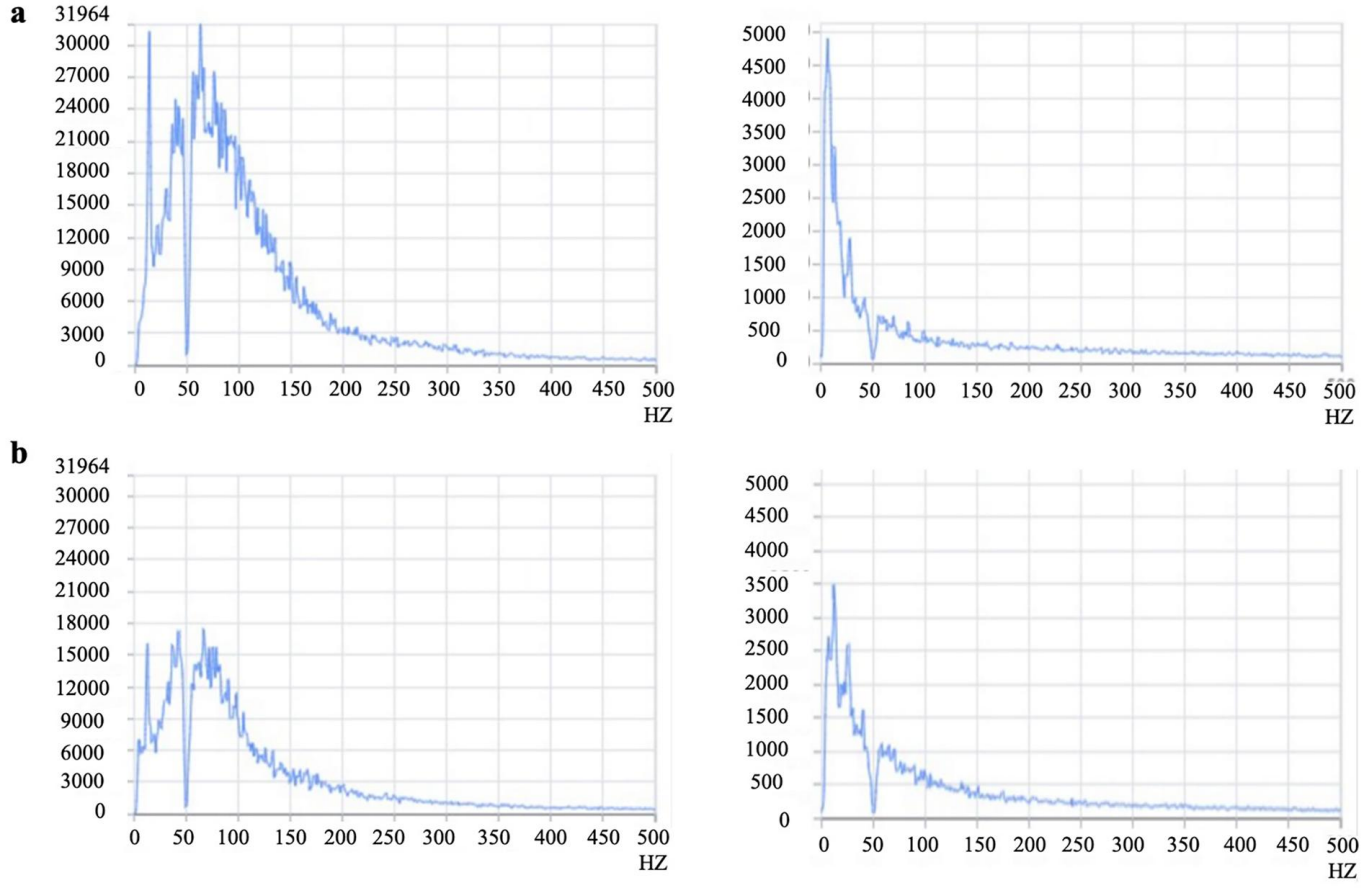
Compares sEMG signals between the normal and abnormal lordosis groups. The panel **a** presents spectrograms of the left SCM during cervical flexion and the left UT during extension in the normal lordosis group, while **b** displays corresponding spectrograms for the left SCM during flexion and left UT during extension in the abnormal lordosis group. UT, upper trapezius; SCM, sternocleidomastoid.

sEMG data were acquired using the DENo wireless sEMG analysis system SG-800B (DENo Medical Technology Co., Ltd., Zhejiang, China). Participants maintained a standardized seated position with relaxed upper limbs naturally adducted. Testing occurred under controlled ambient conditions: temperature 22°C–24°C, relative humidity 40% (27), with isolation from environmental noise, electromagnetic interference, and thermal fluctuations. Skin preparation included degreasing and exfoliation at electrode sites to enhance conductivity, followed by conductive gel application to reduce impedance and optimize signal quality. The UT electrode was positioned at the midpoint between the C7 spinous process and acromion, while the SCM electrode was placed at the middle-inferior third of the line connecting the sternal notch to the mastoid process (28, 29). Muscle activation signals during six-directional cervical motion were recorded at amplitudes ranging from 0 to 5,000 μV and frequencies of 30–350 Hz, with linear signal processing for time-domain and frequency-domain analyses. Acquired sEMG parameters included MPF, MPFs, MF, and MFs. Representative raw sEMG signals from normal and abnormal groups are illustrated in Figure 3.

### 2.5 Statistical analysis

Statistical analyses were performed using SPSS version 27.0 (MacOS; IBM Corp., Armonk, NY, USA). Normality of variables was assessed via the Shapiro–Wilk test. Depending on distribution characteristics, both parametric and non-parametric tests were implemented. Descriptive statistics are presented as mean ± standard deviation (SD) for normally distributed data or median (minimum, maximum) for non-normally distributed data. Independent samples *t*-tests were applied for normally distributed data, while Mann–Whitney *U*-tests were used for non-normally distributed data in between-group comparisons. Relationships with sEMG parameters were examined using Pearson's product-moment correlation coefficients. Statistical significance was set at *p* < 0.05, with results reported as mean ± SD or median (minimum, maximum).

## 3 Results

### 3.1 Baseline characteristics of study participants

The study enrolled 49 participants stratified into two groups: the Abnormal CL Group (Abnormal group, *n* = 25 CNP patients) and the Normal CL Group (Normal group, *n* = 24 healthy participants). The Abnormal group comprised 8 males and 17 females (age 30.29 ± 12.82 years, BMI 22.25 ± 2.95 kg/m^2^), while the Normal group consisted of 3 males and 21 females (age 26.33 ± 10.34 years, BMI 21.50 ± 4.04 kg/m^2^). No statistically significant differences were observed between groups for sex distribution, age, or BMI (*p* > 0.05; Supplementary Table S1). Radiographic measurements revealed a mean C2–C7 Cobb angle of −12.61° ± 3.34° in the Normal group vs. 13.57° ± 2.51° in the Abnormal group, indicating cervical kyphosis or straightened curvature. A statistically significant intergroup difference in C2–C7 Cobb angles was confirmed (*p* < 0.05; Table 1).

**Table 1:** Intergroup comparison of C2–C7 cobb angles between study groups.

Category	Group	Mean ± SD	Mean difference	*t*-test	
				*t*-value	*p*-value
Cobb angle (°)	Abnormal	13.57 ± 2.51	26.18	−25.347	0.000**
	Normal	−12.61 ± 3.34			

** Indicates *p* < 0.01.

### 3.2 Muscle activation during cervical flexion and extension

During cervical flexion, the Abnormal Lordosis Group exhibited significantly higher MPFs and MFs in bilateral SCM muscles compared to the Normal Lordosis Group (all *p* < 0.05). Specifically, the median (Md) MPFs values were −0.4 (left) and −0.3 (right) in the Abnormal Group vs. −0.75 (left) and −0.6 (right) in the Normal Group. Similarly, the MFs Md were −0.16 (left) and −0.13 (right) in the Abnormal Group compared to Md = −0.5 (left) and Md = −0.32 (right) in the Normal Group (Table 2). During cervical extension, for the left UT, the Abnormal Group demonstrated significantly lower MPF (Md = 110.31) and MF (Md = 67.83) than the Normal Lordosis Group (MPF Md = 136.2; MF Md = 100.66) (*p* < 0.05), while exhibiting significantly higher MFs (Md = 0.34) than the Normal Group (MFs Md = −0.27) (*p* < 0.05) (Table 3). For the right UT, both MPFs (Md = −0.44) and MFs (Md = −0.39) were significantly elevated in the Abnormal Group relative to the Normal Group (MPFs Md = −0.86; MFs Md = −0.77) (all *p* < 0.05) (Table 3).

**Table 2:** Between-Group comparison of sEMG parameters during cervical flexion.

Category	Group	Median (Min, Max)	Mean difference	Mann–Whitney *U*-test	
				*z*-value	*p*-value
Flexion SCM-left					
MPF	Abnormal	122.860 (118.0, 130.1)	−13.500	1.651	0.099
	Normal	136.360 (119.6, 144.4)			
MPFs	Abnormal	−0.400 (−0.7, −0.1)	0.350	2.502	0.012*
	Normal	−0.750 (−1.1, −0.6)			
MF	Abnormal	95.090 (85.4, 98.8)	−7.300	1.269	0.205
	Normal	102.390 (94.2, 111.4)			
MFs	Abnormal	−0.160 (−0.4, 0.0)	0.340	2.833	0.005**
	Normal	−0.500 (−0.8, −0.3)			
Flexion SCM-right					
MPF	Abnormal	125.920 (113.2, 129.8)	−0.810	1.547	0.122
	Normal	126.730 (116.7, 137.4)			
MPFs	Abnormal	−0.300 (−0.4, −0.1)	0.300	2.503	0.012*
	Normal	−0.600 (−1.1, −0.2)			
MF	Abnormal	90.720 (85.0, 99.6)	−7.750	1.477	0.140
	Normal	98.470 (85.4, 101.6)			
MFs	Abnormal	−0.130 (−0.3, 0.3)	0.190	2.902	0.004**
	Normal	−0.320 (−0.7, −0.1)			

SCM, sternocleidomastoid; MPF, mean power frequency; MPFs, MPF slopes; MF, median frequency; MFs, MF slopes.

* Indicates statistical significance (*p* < 0.05).

** Indicates *p* < 0.01.

**Table 3:** Between-Group comparison of sEMG parameters during cervical extension.

Category	Group	Median (Min, Max)	Mean difference	Mann–Whitney *U*-test	
				*z*-value	*p*-value
Extension UT-left					
MPF	Abnormal	110.310 (94.0, 123.6)	−25.890	2.450	0.014*
	Normal	136.200 (125.2, 161.1)			
MPFs	Abnormal	0.170 (−0.2, 0.5)	0.680	1.825	0.068
	Normal	−0.510 (−0.7, 0.4)			
MF	Abnormal	67.830 (54.2, 77.8)	−32.830	2.728	0.006**
	Normal	100.660 (78.5, 122.2)			
MFs	Abnormal	0.340 (−0.3, 0.6)	0.610	2.381	0.017*
	Normal	−0.270 (−0.7, 0.0)			
Extension UT-right					
MPF	Abnormal	158.640 (131.3, 175.2)	−5.640	1.651	0.099
	Normal	164.280 (156.3, 185.9)			
MPFs	Abnormal	−0.440 (−0.8, −0.1)	0.420	2.589	0.010**
	Normal	−0.860 (−1.3, −0.4)			
MF	Abnormal	111.210 (87.9, 130.7)	−14.040	1.721	0.085
	Normal	125.250 (105.5, 148.7)			
MFs	Abnormal	−0.390 (−0.6, 0.2)	0.380	2.694	0.007**
	Normal	−0.770 (−1.2, −0.3)			

UT, upper trapezius; MPF, mean power frequency; MPFs, MPF slopes; MF, median frequency; MFs, MF slopes.

* Indicates statistical significance (*p* < 0.05).

** Indicates *p* < 0.01.

### 3.3 SCM and UT muscle activation during cervical lateral flexion

During cervical lateral flexion, outcomes demonstrated asymmetry between left- and right-side movements. During right lateral flexion, the MFs (Md = 0.03) of the right SCM in the Abnormal Lordosis Group was significantly elevated compared to the Normal Lordosis Group (MFs Md = −0.33) (*p* < 0.05). Concurrently, the MF (Md = 89.29) of the right UT was significantly lower in the Abnormal Group vs. the Normal Group (MF Md = 104.29) (*p* < 0.05) (Table 4). In contrast, no statistically significant differences were observed in any electromyographic parameters of the left SCM or left UT during left lateral flexion (Supplementary Table S2).

**Table 4:** Between-group comparison of sEMG parameters during right lateral flexion.

Category	Group	Median (Min, Max)	Mean difference	Mann–Whitney *U*-test	
				*z*-value	*p*-value
Right flexion SCM-right					
MPF	Abnormal	123.400 (118.0, 138.8)	−3.640	0.295	0.768
	Normal	127.040 (120.4, 130.3)			
MPFs	Abnormal	−0.180 (−0.4, 0.0)	0.190	1.390	0.164
	Normal	−0.370 (−0.6, −0.3)			
MF	Abnormal	90.270 (82.6, 101.6)	−2.360	0.817	0.414
	Normal	92.630 (83.3, 103.5)			
MFs	Abnormal	0.030 (−0.2, 0.3)	0.360	2.208	0.027*
	Normal	−0.330 (−0.4, −0.1)			
Right flexion UT-right					
MPF	Abnormal	133.830 (107.7, 155.9)	−4.820	1.512	0.131
	Normal	138.650 (131.0, 159.6)			
MPFs	Abnormal	−0.280 (−1.0, 0.3)	0.200	0.886	0.375
	Normal	−0.480 (−1.0, −0.2)			
MF	Abnormal	89.290 (73.7, 115.4)	−15.000	2.138	0.033*
	Normal	104.290 (91.0, 126.7)			
MFs	Abnormal	−0.210 (−0.6, 0.3)	0.320	1.269	0.205
	Normal	−0.530 (−1.1, 0.0)			

SCM, sternocleidomastoid; UT, upper trapezius; MPF, mean power frequency; MPFs, MPF slopes; MF, median frequency; MFs, MF slopes.

* Indicates statistical significance (*p* < 0.05).

### 3.4 SCM muscle activation during cervical rotation

During cervical rotation to the left, the Abnormal Lordosis Group exhibited significantly lower MPF (Md = 116.62) and MF (Md = 82.34) in the right SCM compared to the Normal Lordosis Group (MPF Md = 123.12; MF Md = 90.17) (*p* < 0.05). Conversely, MPFs (Md = 0.22) and MFs (Md = −0.04) were significantly elevated in the Abnormal Group relative to the Normal Group (MPFs Md = 0.66; MFs Md = −0.29) (*p* < 0.05) (Table 5). During right rotation, only the MPFs of the left SCM was significantly higher in the Abnormal Group (Md = 0.29) vs. the Normal Group (Md = 0.52) (*p* < 0.05), with no statistically significant differences observed in other electromyographic parameters between groups (Supplementary Table S3).

**Table 5:** Between-group comparison of sEMG parameters during left rotation.

Category	Group	Median (Min, Max)	Mean difference	Mann–Whitney *U*-test	
				*z*-value	*p*-value
Left rotation SCM-right					
MPF	Abnormal	116.620 (107.0, 125.2)	−6.500	2.033	0.042*
	Normal	123.120 (115.7, 134.3)			
MPFs	Abnormal	−0.220 (−0.4, 0.1)	0.440	2.485	0.013*
	Normal	−0.660 (−0.8, 0.0)			
MF	Abnormal	82.340 (75.1, 94.1)	−7.830	2.033	0.042*
	Normal	90.170 (82.0, 103.8)			
MFs	Abnormal	−0.040 (−0.2, 0.1)	0.250	2.172	0.030*
	Normal	−0.290 (−0.5, 0.0)			

SCM, sternocleidomastoid; MPF, mean power frequency; MPFs, MPF slopes; MF, median frequency; MFs, MF slopes.

* Indicates statistical significance (*p* < 0.05).

### 3.5 Correlation between muscle fatigue levels and the C2–C7 cobb angle

**Figure 4:**
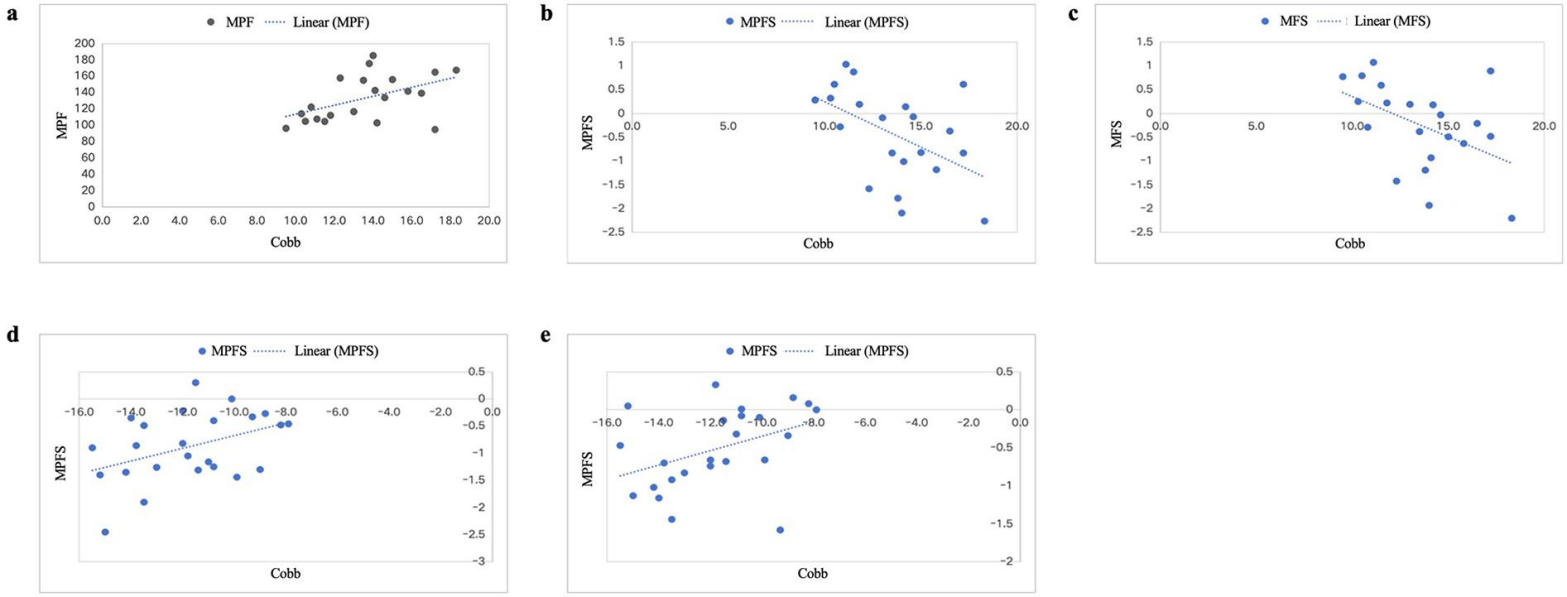
This shows the correlation between the C2–C7 cobb angle and the sEMG parameters. **(a–c)** Present correlational analyses for the Abnormal Lordosis Group, with identical descriptions for **(a–c)** showing the right UT during right lateral flexion. Panels d and e display correlations for the Normal Lordosis Group, depicting the right UT during extension **(d)** and the right SCM during left rotation **(e)**, respectively. UT, upper trapezius; SCM, sternocleidomastoid.

Within the Abnormal Lordosis Group, the C2–C7 Cobb angle demonstrated weak correlations with electrophysiological parameters of the right UT during right lateral flexion, including MPF (*r* = 0.492, *p* < 0.05), MPFs (*r* = −0.496, *p* < 0.05), and MFs (*r* = −0.468, *p* < 0.05). In the Normal Lordosis Group, weak positive correlations were observed between the C2–C7 Cobb angle and MPFs of the right UT during extension (*r* = 0.415, *p* < 0.05), as well as MPFs of the right SCM during left rotation (*r* = 0.407, *p* < 0.05) (Figure 4).

## 4 Discussion

This study utilized multi-directional dynamic sEMG to investigate muscle fatigue characteristics associated with abnormal CL, revealing three primary findings: First, patients with abnormal cervical curvature exhibited significantly higher levels of neck muscle fatigue during dynamic activities compared to those with normal curvature. Second, based on anatomical location and functional differences, the UT and SCM demonstrated specific fatigue patterns under abnormal curvature. Third, a correlation exists between the cervical Cobb angle and the degree of muscle fatigue.

### 4.1 Divergent fatigue patterns in UT and SCM muscles

This study confirms that abnormal cervical curvature significantly exacerbates cervical muscle fatigue. This finding supports previous perspectives suggesting that non-structural misalignments in the cervical sagittal plane impact the physiological load on the musculoskeletal system (30). However, this effect manifested differently in the UT and SCM, which may be attributed to their distinct anatomical and functional roles. The UT fatigue mechanism may exhibit a bidirectional relationship with abnormal cervical curvature: Cervical kyphosis shortens the distance between the UT origin and insertion points (C1-C6 spinous processes to the lateral clavicle) (28), leading to reduced muscle fiber resting length. This may force the muscle to operate at an unfavorable length-tension relationship, reducing maximal force generation. Furthermore, increased scapular downward rotation disrupts UT-serratus anterior synergy, elevating energy demand, while reduced cervical curvature alters zygapophyseal joints stress, triggering a proprioceptive-mediated UT spasm and a self-perpetuating cycle of “curvature abnormality - stress concentration - abnormally increased muscle tension”. These mechanisms align with reported trapezius/rhomboid inhibition, UT overactivation (31), and impaired muscle function (32). A recent sEMG analysis of the UT in patients with neck pain further indicated that short-duration sustained muscle activity (e.g., ≤60 s) was positively correlated with neck pain, whereas prolonged sustained muscle activity (>10 min) demonstrated an inverse relationship (33). Of note, a cross-sectional study revealed that the level of UT muscle activity was significantly positively correlated with the degree of neck dysfunction in CNP (34). Thus, UT activity intensity and duration contribute to fatigue-induced exacerbation of neck pain.

In contrast, SCM fatigue appears to involve more complex neuromuscular adaptations beyond pure mechanical overload. The SCM is rich in muscle spindle proprioceptors (35), and its fatigue impacts sensorimotor integration processes in the cerebellum and motor cortex (36). This sensorimotor deficit may lead to compensatory over-reliance on the SCM while inhibiting deeper stabilizers, perpetuating a cycle of fatigue (37). Notably, his disruption in proprioceptive input further impairs the central nervous system's ability to regulate cervical position, leading to postural control disturbance and thereby exacerbating SCM fatigue. In addition, the SCM receives bilateral corticobulbar inputs and participates in head posture regulation through anticipatory postural adjustments prior to movement onset (38). However, under conditions of predictable or self-induced perturbations, the activation amplitude of SCM is significantly reduced (39, 40). This suggests that the reflexive activation of SCM is posture-dependent and modulated by task-specific postural demands (41). Altered or poor posture may consequently induce dysregulated SCM activation, thereby promoting the development of fatigue. This provides a new perspective for understanding postural compensatory mechanisms in CNP.

### 4.2 The curvature-fatigue relationship

A pivotal finding is the complex relationship between the C2–C7 Cobb angle and sEMG-derived fatigue parameters. Within the abnormal curvature group, a decreasing Cobb angle (worsening kyphosis) was paradoxically associated with a relative reduction in fatigue levels according to certain sEMG metrics. Conversely, within the normal group, appropriate lordosis increase (increasing Cobb angle) correlated with reduced risk of muscle fatigue. However, it is crucial to emphasize that although kyphosis within the abnormal group might be associated with reduced muscle fatigue, long-term kyphosis carries risks including reduced cervical stability, joint degeneration, and nerve compression (42). This underscores that the relationship between spinal alignment and muscle function is not monolithic and is also influenced by the spinal curvature.

### 4.3 Clinical implications

This study suggests CL may serve as an important clinical parameter for assessing neck muscle function. However, the clinical significance of CL remains debated in previous research: a large-scale MRI follow-up study found no significant association between CL and clinical symptoms (43); whereas other studies identified increased thoracic inlet angle as an independent predictor of CNP (44), elevated T1S angle associated with cervical disc degeneration (45), and a low T1S-CL angle combined with a high C2-C7 SVA predicting neck dysfunction (46). We posit this controversy may partly stem from insufficient standardization in research methodologies (e.g., arm position, gaze direction) and from viewing the cervical spine merely as an extension of the thoracolumbar spine, overlooking the pivotal biomechanical role of the craniocervical-thoracic junction and associated shoulder girdle musculature. Simultaneously, our findings underscore the importance of incorporating muscle functional parameters (e.g., dynamic fatigue characteristics) into the assessment framework.

### 4.4 Limitations and future research

This study has limitations: no subgroup analysis was conducted for straightened vs. kyphosis curvature; the influence of participant handedness on muscle activation was not addressed; potential confounding effects from occupational postures were not strictly controlled; enrolled CNP patients had their Visual Analogue Scale scores controlled below 6. Future research should expand the sample size and conduct sub-group regression analyses, integrate musculoskeletal ultrasonography for dynamic observation of muscle fiber morphological changes, and establish prospective cohorts to track the long-term relationship between cervical curvature progression and muscle function evolution.

## 5 Conclusion

This study confirms that abnormal cervical physiological curvature significantly exacerbates neck muscle fatigue during dynamic activities. Correlation analysis revealed that within the abnormal curvature group, increasing kyphosis severity was associated with reduced muscle fatigue levels, whereas within the normal curvature group, increasing lordosis was significantly associated with reduced fatigue risk. This validates the hypothesis that abnormal curvature triggers compensatory muscle activation, suggesting a need for stratified clinical interventions: patients with kyphosis should be monitored for long-term degeneration risks, while those with insufficient lordosis may benefit from strengthening deep muscle training. By being the first to integrate Cobb angle assessment with multi-directional dynamic sEMG analysis, this study provides a biomechanical basis for informing rehabilitation strategies.

## Data Availability

The original contributions presented in the study are included in the article/Supplementary Material, further inquiries can be directed to the corresponding author.
