# Supplementary material for: Biomechanical mechanisms of multidirectional dynamic compensatory muscle fatigue induced by abnormal cervical curvature: a cross-sectional case-control study based on surface electromyography and Cobb angle
Source: Front Sports Act Living. 2025 Dec 4;7:1704956. doi: 10.3389/fspor.2025.1704956 (PMC12711822; doi:10.3389/fspor.2025.1704956)
Supplement: Supplementary file 1 [file Table1.docx]

**SUPPLEMENTARY TABLE 1** **Statistical analysis of baseline characteristics of study participants.**

| **Feature** | **Abnormal Group** | **Normal Group** | **Test Statistic** | ***p-*value** |
| --- | --- | --- | --- | --- |
| Sex (male/female) | 8 / 17 | 3 / 21 | *x^2^* = 1.212 | 0.271 |
| Age (years) | 30.29 ± 12.82 | 26.33 ± 10.34 | *t* = 1.103 | 0.283 |
| BMI (kg/m^2^) | 22.25 ± 2.95 | 21.50 ± 4.04 | *t* = 0.567 | 0.577 |

**SUPPLEMENTARY TABLE 2 Between-Group comparison of sEMG parameters during left lateral flexion.**

| **Category** | **Group** | **Median (Min, Max)** | **Mean Difference** | **Mann-Whitney U test** | |
| --- | --- | --- | --- | --- | --- |
|  |  |  |  | ***z*-value** | ***p*-value** |
| **Left flexion SCM-left** | | | | | |
| MPF | Abnormal | 118.080 (111.3, 136.2) | -17.440 | 1.338 | 0.181 |
|  | Normal | 135.520 (118.9, 145.9) |  |  |  |
| MPFs | Abnormal | -0.090 (-0.7, 0.1) | 0.260 | 1.686 | 0.092 |
|  | Normal | -0.350 (-0.9, -0.1) |  |  |  |
| MF | Abnormal | 88.120 (81.3, 103.0) | -10.610 | 1.477 | 0.140 |
|  | Normal | 98.730 (84.2, 104.7) |  |  |  |
| MFs | Abnormal | 0.040 (-0.4, 0.3) | 0.210 | 1.460 | 0.144 |
|  | Normal | -0.170 (-0.6, 0.1) |  |  |  |
| **Left flexion UT-left** | | | | | |
| MPF | Abnormal | 92.240 (80.1, 112.0) | -15.490 | 1.686 | 0.092 |
|  | Normal | 107.730 (88.0, 132.2) |  |  |  |
| MPFs | Abnormal | 0.080 (-0.6, 0.3) | 0.340 | 1.060 | 0.289 |
|  | Normal | -0.260 (-1.1, 0.2) |  |  |  |
| MF | Abnormal | 57.970 (51.2, 76.8) | -12.010 | 0.991 | 0.322 |
|  | Normal | 69.980 (54.8, 92.8) |  |  |  |
| MFs | Abnormal | 0.090 (-0.2, 0.5) | 0.180 | 1.164 | 0.244 |
|  | Normal | -0.090 (-0.9, 0.2) |  |  |  |

SCM, sternocleidomastoid; UT, upper trapezius; MPF, mean power frequency; MPFs, MPF slopes; MF, median frequency; MFs, MF slopes.

**SUPPLEMENTARY TABLE 3 Between-Group comparison of sEMG parameters during right rotation.**

| **Category** | **Group** | **Median (Min, Max)** | **Mean Difference** | **Mann-Whitney U test** | |
| --- | --- | --- | --- | --- | --- |
|  |  |  |  | ***z*-value** | ***p*-value** |
| **Right rotation SCM-left** | | | | | |
| MPF | Abnormal | 118.320 (112.7, 130.7) | -8.490 | 1.825 | 0.068 |
|  | Normal | 126.810 (118.9, 140.4) |  |  |  |
| MPFs | Abnormal | -0.290 (-0.4, 0.0) | 0.230 | 2.312 | 0.021* |
|  | Normal | -0.520 (-0.7, -0.1) |  |  |  |
| MF | Abnormal | 88.050 (76.5, 96.8) | -11.570 | 1.894 | 0.058 |
|  | Normal | 99.620 (84.7, 109.4) |  |  |  |
| MFs | Abnormal | -0.030 (-0.4, 0.3) | 0.340 | 1.651 | 0.099 |
|  | Normal | -0.370 (-0.6, 0.1) |  |  |  |

SCM, sternocleidomastoid; MPF, mean power frequency; MPFs, MPF slopes; MF, median frequency; MFs, MF slopes.

Note: * indicates statistical significance (*p* < 0.05).
