# Supplementary material for: Biomechanical mechanisms of multidirectional dynamic compensatory muscle fatigue induced by abnormal cervical curvature: a cross-sectional case-control study based on surface electromyography and Cobb angle
Source: Front Sports Act Living. 2025 Dec 4;7:1704956. doi: 10.3389/fspor.2025.1704956 (PMC12711822; doi:10.3389/fspor.2025.1704956)
Supplement: Supplementary file 2 [file Datasheet1.docx]

This study recruited participants comprising chronic neck pain patients and healthy volunteers, with two distinct inclusion criteria sets established for each cohort and a unified set of exclusion criteria.

- **Inclusion Criteria**

**Chronic Neck Pain Patients**

​1. Age 18-65 years;

​2. Body mass index (BMI) 18-35 kg/m^2^;

​3. Dynamic lateral cervical radiographs demonstrating C2–C7 Cobb angle > −4° (indicating straightened/kyphotic cervical curvature);

​4. Neck pain duration 3–6 months, Visual Analogue Scale (VAS) score < 6, and clinical diagnosis of chronic neck pain;

​5. Ability to actively participate and cooperate with experimental procedures.

**Healthy Volunteers**

​1. Age 18–65 years;

​2. BMI 18–35 kg/m²;

​3. Dynamic lateral cervical radiographs showing C2–C7 Cobb angle between −16° and −4° (indicating normal cervical lordosis);

​4. Absence of neck pain or cervical discomfort;

​5. Voluntary participation in the study.

- **Exclusion Criteria**

​1. Cognitive impairment precluding cooperation with assessments;

​2. Neuromuscular disorders, sarcopenia, or other conditions affecting muscle function evaluation;

​3. Unstable vital signs or cachexia;

​4. Neck VAS score > 6;

​5. Cervical spondylotic myelopathy or cervical instability;

​6. Severe intervertebral disc pathology;

​7. Refusal or unwillingness to participate.
